# Supplementary material for: Exopolysaccharide from Porphyridium cruentum (purpureum) is Not Toxic and Stimulates Immune Response against Vibriosis: The Assessment Using Zebrafish and White Shrimp Litopenaeus vannamei
Source: Mar Drugs. 2021 Feb 28;19(3):133. doi: 10.3390/md19030133 (PMC7997376; doi:10.3390/md19030133)
Supplement: Supplementary file 1 [file marinedrugs-19-00133-s001.pdf]

Supplementary File

Table S1. Toxicity test using the ZET exposed in the different concentration of extracellular polysaccharide (EPS, sPS) and exposure time of 24, 48, 72 and 96 h respectively. The toxicity is based as the number of zebrafish embrionic mortality (Hpf).

**Concentration**	**Replication**	**Number of embrio/plate**	**Time (h)**			
			24	48	72	96
**0%**	1	20	0	1	0	0
	2	20	3	1	0	1
	3	20	4	0	0	1
	average		2.33	0.67	0.00	0.67
	SD		2.08	0.58	0.00	0.58
**5%**	1	20	0	1	0	0
	2	20	1	1	0	0
	3	20	1	1	0	0
	average		0.67	1.00	0.00	0.00
	SD		0.58	0.00	0.00	0.00
**10%**	1	20	3	1	0	0
	2	20	4	1	1	0
	3	20	3	1	0	0
	average		3.33	1.00	0.33	0.00
	SD		0.58	0.00	0.58	0.00
**15%**	1	20	7	4	0	6
	2	20	8	4	0	7
	3	20	3	2	0	10
	average		6.00	3.33	0.00	7.67
	SD		2.65	1.15	0.00	2.08
**20%**	1	20	9	1	0	8
	2	20	7	2	1	8
	3	20	14	1	0	5
	average		10.00	1.33	0.33	7.00
	SD		3.61	0.58	0.58	1.73
